# The Old Order Changeth

**Published:** 1920-12-11

**Authors:** 


### THE OLD ORDER CHANGETH.

In my quest for information I have drawn a blank. The ladies who lit their cigarettes with the College voting papers, and likewise those who did not, apparently have arrived at the conclusion that the time for contemplation has come, and, either that there is no use in saying or doing, or else that there is no necessity. As for Lord Knutsford, he probably realises that he has already said more than was wise. He shrinks from elaborating the argument that the College is too young, or from justifying his office of councillor. The only correspondent who has vouchsafed me a reply is a lady whose outlook is eminently conservative and conventional. Nurses, she observes, have received what they most desired, a State Bill of Registration, and "therefore it seems to me unnecessary that they should at the present time be before the House of Commons. They have the General Nursing Council, also the Council of the College of Nursing, and to these two representative bodies the nurses can look to further their interests. No doubt when the General Nursing Council really gets to work it will do much to help its members." And other words to the same effect.

### The General Nursing Council.

This is not the first reference that I have seen to the General Nursing Council by persons who have an erroneous idea of its functions. The General Nursing Council is not a militant body, nor have they any other than a purely academic duty to perform. This Council is a Committee to watch the working of the Registration Act, to see that a proper examination standard is established and maintained, and that other conditions of registration are in the public interest and in accord with the wishes of the majority of the profession. They hold no commission or warrant to justify them in advocating the economic claims of nurses, and therefore those who are counting upon their activity in this field will be disappointed.

### The Significance of Silence.

The silence of the nurse, especially at such a crisis, is a phenomenon which I hope will be investigated. It is not a proof that interest in the College is dead, but it is in accord with such a possibility. Failure to vote at the annual election of representatives, with all the documents put into their possession, was a distinct indication of wilful negligence. Failure to respond to a discussion of College action and responsibilities merely emphasises their attitude of inertia. And yet it is undeniable that at this moment the trained nurse stands at the parting of the ways. The past five years have witnessed stupendous changes. Empires have been shattered and proud dynasties suddenly and cruelly terminated; noble battleships, the glory of their builders, have been sold as scrap iron. That which was last is first, and first last.

Thus times do shift; each thing his turne does hold;
New things succeed, as former things grow old.

It appears to me that even in respect of nursing it will be found ere long that the old order changeth. The probationary system is rocking, and, as I pointed out last week, some institutions are already introducing schemes by which nurses will be produced without conforming with the orthodox method. If hospitals are thrown on the rates the old order will automatically disappear, and with this change the old brigade of nurses will become obsolescent.

Mark you, I am not prophesying, but merely chronicling the initial stage of a movement already in progress, and I see no outstanding personality, nor any representative society, engaged in doing battle for what I call the old brigade. My translation of Lord Knutsford's remarks at the College meeting is that that institution is non-militant, non-combatant. That it is "too young" is a euphemism, signifying politely that concerning fighting there is nothing doing.

### The Battle.

The nurse had, and still has, a strong case, but one which could be won only by fighting. She had a claim on the nation for recognition of her life's service, offered with supreme devotion. The war proved her worth, for whether by land or sea, wherever there was death or danger, there was the nurse. And yet when the war ended, and reconstruction succeeded, the first to scrap her claim was, if you please, the Minister of Health. The conditions laid down for qualifying as health visitors are such that only women of considerable means can avail of them, and no nurse can do so without surrendering her occupation. And yet a trained nurse is within hailing distance of being admirably qualified.

This monstrous injustice was allowed to pass unchallenged when all England should have been shouting shame. My own impression is that the nurse expected the College militant to take up her cause. She had, so to speak, employed a solicitor, and handed over to him the task of conducting her affairs. But, apparently, the function of the College is non-militant, and consists of promoting the nurse's social and intellectual welfare—a very useful function if there was as well a battery of artillery. The Y.M.C.A. was magnificent behind the lines, but the men-at-arms it was who won the war.

When the College of Nursing was called into being by Sir Arthur Stanley, I formed a very distinct picture in my mind of what it might, and in all probability would, accomplish. I venture to believe that mine was a reasonable concept, and, with the Editor's permission, I hope to present this to The Hospital readers for their judgment.

IERNE.

